# Medication adherence in randomized controlled trials evaluating cardiovascular or mortality outcomes in dialysis patients: A systematic review

**DOI:** 10.1186/s12882-017-0449-1

**Published:** 2017-01-31

**Authors:** Karumathil M Murali, Judy Mullan, Jenny H. C. Chen, Steven Roodenrys, Maureen Lonergan

**Affiliations:** 1 Department of Nephrology, Wollongong Hospital, Wollongong, NSW 2500 Australia; 2 Graduate School of Medicine, University of Wollongong, Wollongong, NSW Australia; 3 School of Psychology, University of Wollongong, Wollongong, NSW Australia

**Keywords:** Cardiovascular outcomes, Dialysis, Medication adherence, Study drug discontinuation, Mortality, Randomized controlled trials

## Abstract

**Background:**

Medication non-adherence is common among renal dialysis patients. High degrees of non-adherence in randomized controlled trials (RCTs) can lead to failure to detect a true treatment effect. Cardio-protective pharmacological interventions have shown no consistent benefit in RCTs involving dialysis patients. Whether non-adherence contributes to this lack of efficacy is unknown. We aimed to investigate how medication adherence and drug discontinuation were assessed, reported and addressed in RCTs, evaluating cardiovascular or mortality outcomes in dialysis patients.

**Methods:**

Electronic database searches were performed in MEDLINE, EMBASE & Cochrane CENTRAL for RCTs published between 2005–2015, evaluating self-administered medications, in adult dialysis patients, which reported clinical cardiovascular or mortality endpoints, as primary or secondary outcomes. Study characteristics, outcomes, methods of measuring and reporting adherence, and data on study drug discontinuation were analyzed.

**Results:**

Of the 642 RCTs in dialysis patients, 22 trials (12 placebo controlled), which included 19,322 patients, were eligible. The trialed pharmacological interventions included anti-hypertensives, phosphate binders, lipid-lowering therapy, cardio-vascular medications, homocysteine lowering therapy, fish oil and calcimimetics. Medication adherence was reported in five trials with a mean of 81% (range: 65–92%) in the intervention arm and 84.5% (range: 82–87%) in the control arm. All the trials that reported adherence yielded negative study outcomes for the intervention. Study-drug discontinuation was reported in 21 trials (mean 33.2%; 95% CI, 22.0 to 44.5, in intervention and 28.8%; 95% CI, 16.8 to 40.8, in control). Trials with more than 20% study drug discontinuation, more often yielded negative study outcomes (*p* = 0.018). Non-adherence was included as a contributor to drug discontinuation in some studies, but the causes of discontinuation were not reported consistently between studies, and non-adherence was listed under different categories, thereby potentiating the misclassification of adherence.

**Conclusions:**

Reporting of medication adherence and study-drug discontinuation in RCTs investigating cardiovascular or mortality endpoints in dialysis patients are inconsistent, making it difficult to compare studies and evaluate their impact on outcomes. Recommendations for consistent reporting of non-adherence and causes of drug discontinuation in RCTs will therefore help to assess their impact on clinical outcomes.

**Electronic supplementary material:**

The online version of this article (doi:10.1186/s12882-017-0449-1) contains supplementary material, which is available to authorized users.

## Background

Poor adherence to treatment is an important problem in the management of chronic diseases [1]. Non-adherence is widely prevalent, yet, frequently under-recognized and is associated with higher mortality and morbidity, as well as increased treatment costs [2]. Non-adherence is multi-dimensional and determined by five major interacting domains; namely socio-economic, health care system related, therapy related, disease related and patient related factors [1]. Poor socio-economic status, complexity of treatment regimen, poor health literacy and comorbidities (such as depression and cognitive impairment) predispose patients with end stage kidney disease (ESKD) on dialysis to become non-adherent with their medications [3, 4]. The problem can be particularly challenging, when poor adherence doesn’t have any noticeable short-term effect on symptoms [5]. Poor treatment adherence is predictive of increased mortality among dialysis patients [4], but the reporting of adherence in clinical trials involving dialysis patients is inconsistent.

Cardiovascular mortality is 10–20 fold greater in dialysis patients, compared to age and sex-matched controls without chronic kidney disease (CKD) [6]. Traditional risk factors account for up to 50% of cardiovascular disease in CKD [7], while non-traditional factors unique to renal disease, like anemia, disordered bone mineral metabolism and oxidative stress, also contribute to poor cardiovascular outcomes. Trials evaluating cholesterol lowering medications like HMG-CoA reductase inhibitors, which have proven efficacy in reducing cardiovascular outcomes in the general population, have shown no significant benefits in patient on dialysis [8–11]. Modification of the risk factors like correction of anaemia [12], homocystine lowering therapies [13], treatment with omega-3 fatty acids [14], control of hyperphosphataemia [15, 16], and treatment of secondary hyperparathyroidism [17] have also shown no consistent benefit in improving cardiovascular mortality or significant clinical events in dialysis patients. To explain this lack of efficacy of cardio-protective pharmacological interventions, it has been suggested that the pathogenesis of cardiovascular disease in ESKD, might be different from that in the general population, making it less amenable to interventions [6]. Whether poor medication adherence contributes to the lack of efficacy of these pharmacological interventions is unknown. In clinical trial settings, a high frequency of non-adherence (i.e. failure to adhere to prescribed treatments) can result in failure to detect a true difference, due to the loss of statistical power [18]. In addition, a high frequency of study drug discontinuation, which can be due to poor treatment adherence as well as several factors, such as adverse events, drop-out from the study or withdrawal due to protocol specified events like kidney transplantation, can also lead to a false negative study outcome due to loss of statistical power. Consistent reporting of the causes of drug discontinuation is needed to compare studies with respect to the contribution of non-adherence to discontinuation and evaluate their impact on clinical outcomes.

In this review, we sought to examine whether the important issue of adherence to prescribed treatment and study drug discontinuation were adequately and consistently assessed, reported and appropriately addressed in the randomized clinical trials (RCTs) evaluating self-administered cardioprotective medications compared to controls (placebo, another active medication or usual care) in improving cardiovascular or mortality outcomes in patients undergoing dialysis.

## Methods

We included all RCTs published as full-text journal articles, over a ten-year period (2005–2015) in this systematic review. The time period was chosen because of the improved awareness of the need to monitor medication adherence in clinical outcomes of intervention trials in recent years. The studies that investigated the effect of any self-administered pharmacological treatment in ESKD patients undergoing dialysis, and reported clinical cardiovascular events or mortality, as the pre-specified primary or secondary outcomes were included.

### a. Search strategy

Electronic database searches were performed in MEDLINE, EMBASE and Cochrane CENTRAL register of controlled trials for articles published in English, from 2005 onwards using standard search strategies. Medical subject headings included: ‘clinical trial’, ‘trial’, ‘randomized trial’, ‘single blind’ or ‘double blind’; ‘cardiovascular disease’, ‘cardiovascular outcome’, or ‘mortality’; and ‘dialysis’, ‘renal dialysis’ or ‘peritoneal dialysis’, with the search limited to between 1st January 2005-31st December 2015. Search results in the form of titles and abstracts were analyzed by two authors (KM, JC) to ensure inter-rater agreement, regarding which studies to include in the final review, based on inclusion criteria outlined below. Any disagreement was resolved by discussion among all authors. References within included articles and other important reviews regarding the topic were hand-searched to identify reports that might have been missed in the previous search.

### b. Study selection criteria and characteristics

Studies published as full-text articles that included ESKD patients undergoing haemodialysis or peritoneal dialysis alone were considered eligible. Trials, that recruited both dialysis and non-dialysis patients were included, only if the article provided information on the mortality or cardiovascular outcomes for the sub-group of participants on dialysis. This review included only trials comparing a self-administered pharmacological intervention to a control therapy (placebo, another active therapy or usual care). The pre-specified primary or secondary outcomes had to report at least one clinical cardiovascular outcome, which could include fatal or non-fatal cardiovascular events, a composite or death due to any cause. Studies reporting surrogate cardiovascular endpoints, including radiological (e.g. vascular calcification) or biochemical markers (e.g. troponin levels) of cardiovascular disease, as the only primary or secondary outcome were excluded.

### c. Data abstraction and synthesis

A standard check-list, for specific data as described below, was used to abstract information from the included studies. Two authors (KM, JC) abstracted data to generate independent datasheets, comprising of quantitative and qualitative information, which were compared to verify inter-rater agreement.

The abstracted data from each study included the: year of publication; journal; first author’s surname; funding source; study acronym; study period; number of participants in the intervention and control arms; study population; inclusion and exclusion criteria; the trialed intervention and control treatments; primary and secondary outcomes; randomization method; information on allocation concealment; blinding of participant, investigator and/or outcome assessment; analysis type (e.g. intention to treat); completeness of outcome data; likelihood of selective reporting; follow-up duration; drop-out rate; whether the study was positive or negative for the outcome of interest; significant secondary outcomes; and reported death from all causes.

We specifically examined whether medication adherence was evaluated, reported and addressed in the included trials. If reported, the method of measuring adherence and its prevalence were assessed. Any method of measurement of individual patient’s adherence was considered acceptable. We recorded the number of subjects discontinuing study medication during the course of the study, reasons for study medication withdrawal and whether non-adherence was identified as a contributor. Where medication adherence was not reported in an article, we contacted the authors to understand whether it was evaluated in the trial. All authors were asked the same question: whether medication adherence was assessed, if yes, what method was used and what was the reported level of adherence.

### d. Statistical methods

Inter-rater reliability was assessed using Cohen’s kappa statistics. The average medication adherence and study drug discontinuation from the included trials were reported as a mean percentage. Proportions were expressed as percentages and Fisher’s exact test was used to compare proportions. The analyses were conducted using Stata® version 12.1.

## Results

### a. Trial flow

**Fig. 1:**
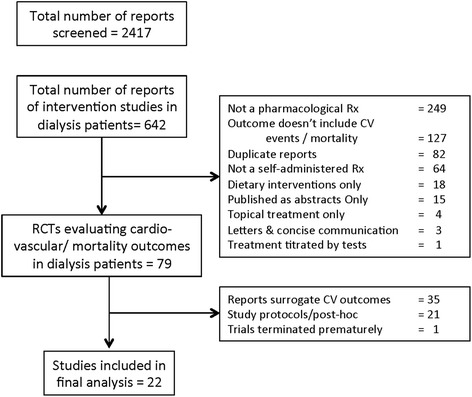
Flow chart showing total number of retrieved citations, reasons for exclusion and the number of studies included in the final analysis

Electronic searches of all three databases returned 2417 reports, and after excluding non-intervention and non-randomized studies, 642 articles were identified. Out of this, 22 trials were included in the final analysis in accordance with the specified inclusion and exclusion criteria (Fig. 1).

### b. Description of studies

Nineteen (86%) trials recruited patients on haemodialysis only, while the remaining trials [9, 11, 19] enrolled dialysis and non-dialysis patients. In this latter group however, separate outcomes data were provided for the dialysis patients.

The pharmacological intervention varied among the 22 included trials. Six studies [20–25] evaluated anti-hypertensive medications, four studies trialed lipid lowering agents [8–11], four studies trialed phosphate-binding drugs [15, 16, 26, 27] three studies trialed cardiovascular medications [28–30] including anti-platelet agents, two studies trialed homocysteine lowering therapy (folic acid) [19, 31], two studies trialed fish oil [14, 32] and one study trialed calcimimetics [17].

Twelve of the 22 studies were double-blind placebo controlled trials, while six trials compared the intervention with usual care as their control, and the remaining four studies tested the intervention against another active agent using an open-label design. Pharmaceutical sponsors were the main source of funding for nine studies, which recruited a total of 15,166 dialysis patients, as compared to the 4,156 patients recruited in the other thirteen studies, funded by non-pharmaceutical sponsors. A comprehensive summary of the 22 trials included in this review is given in Table 1.

**Table 1:** Summary of characteristics, study outcomes and details of addressing and reporting adherence in the randomized controlled trials included in the study

Reference	Year of publication	Population	Total N (Intervention/Control)	Therapy (Intervention/Control)	Trial outcome	Reported Adherence	Addressing Adherence	Drug discontinuation: Intervention/Control arms
Baigent et al. [9]	2011	HD & PD^a^	3023 (1533/1490)^b^	Simvastain + Ezetimibe/Placebo	Negative^b,c^	65%^b^ in the Inter-vention group	Run-in phase to identify non-compliers	33%/36%^e,f^
Block et al. [26]	2007	HD	127 (60/67)	Sevalamer/Ca based binders	Positive	Not reported	Specified as not checked	Not reported
Chertow et al. [17]	2012	HD	3883 (1948/1935)	Cinacalcet/Placebo	Negative	Not reported	TNA reported as cause of therapy discontinuation. Reports lag censored analysis after therapy cessation	67%/71%^f,g,h^
Cice et al. [24]	2010	HD	332 (165/167)	Add-on Telmisartan (to ACEI)/Placebo	Positive	Not reported	Not addressed	16/11%^f,i^
Di Iorio et al. [27]	2013	HD	466 (232/234)	Sevalamer/Ca based binders	Positive	Not reported	Not addressed	14%/15%^i^
Dixon et al. [28]	2009	HD	649 (321/328)	Dipyridamole + Aspirin/Placebo	Negative^d^	83%/83%	Assessed by pill count & reported	30%/26%^f,i^
Fellstrom et al. [10]	2009	HD	2773 (1389/1384)	Rosuvastatin/Placebo	Negative	92%/86%	Assessed by pill count & reported	73%/74%^f,g,h,i,j^
Iseki et al. [25]	2013	HD	469 (235/234)	Olmesartan/Usual care but No ARB or ACEI	Negative	Not reported	Not addressed	49% in intervention^f,h^
Jamison et al. [19]	2007	HD & PD^a^	751 (372/379)^b^	Folic acid + B6 + B12/Placebo	Negative	85%/87%	Assessed by pill count & reported	27%/26%^i^
Lok et al. [32]	2012	HD	196 (99/97)	Fish oil/Placebo	Positive^d^	Not reported	Blood tests supporting drug effect reported, but not at patient level	16%/16%^i,j^
Matsumoto et al. [30]	2014	HD	309 (157/152)	Spironolactone/Usual care	Positive	Not reported	Non-adherent patients excluded from participation	29%/16%^f^
Nishimura et al. [29]	2009	HD	129 (64 + 65)	Nicorandil/Usual care but no Nicorandil	Positive	Not reported	Not addressed	0%/0%
Righetti et al. [31]	2006	HD	88 (37/51)	Folic acid/Usual care	Positive	Not reported	Reported as measured, but not reported	72% ^e,f,h,I,j^
Stegmayr et al. [11]	2005	HD & PD^a^	110 (53/57)^b^	Atorvastatin/Placebo	Negative	Not reported	Non-adherent patients excluded from participation	33%/6%^f^
Suki et al. [15]	2007	HD	2103 (1053/1050)	Sevalamer/Ca based binders	Negative	Not reported	TNA reported as cause of therapy discontinuation	48%/51%^f,g,h^
Suzuki et al. [22]	2008	HD	366 (183/183)	Any ARB/Usual care but no ARB	Positive	Not reported	Not addressed	2%/2%^f^
Svensson et al. [14]	2006	HD	206 (103/103)	Omacor (3-PUFA)/Placebo	Negative	Not reported	Non-adherent patients excluded, Blood tests supporting drug effect reported, but patient level adherence not reported	27%/22%^f,g^
Takahashi et al. [21]	2006	HD	80 (43/37)	Candesartan/Usual care	Positive	Not reported	Non-adherent patients excluded from participation	0%/0%
Tepel et al. [23]	2008	HD	251 (123/128)	Amlodipine/Placebo	Negative	Not reported	TNA reported as cause of therapy discontinuation	63%/65%^f,g,h,i,j^
Wanner et al. [8]	2005	HD	1255 (619/636)	Atorvastatin/Placebo	Negative	80%/82%	Assessed by pill count & reported	23%/24%^f^
Wilson et al. [16]	2009	HD	1354 (680/674)	Lanthanum/Usual care	Negative	Not reported	Not addressed	72%/53%^f,g,h,i,j^
Zannad et al. [20]	2006	HD	397 (196/201)	Fosinopril/Placebo	Negative	Not reported	Not addressed	4%/5%^h^

^a^The trial also included patient not on dialysis. ^b^The data given pertains to dialysis patients only. ^c^The trial was positive for the overall cohort. ^d^Cardiovascular or mortality outcome was a secondary outcome. ^e^The result is for the overall cohort. ^f^Includes adverse events as a cause of drug discontinuation. ^g^Includes non-adherence as a cause of drug discontinuation. ^h^Includes kidney transplantation as a cause of drug discontinuation. ^i^Includes drop-out or loss to follow up as a cause of drug discontinuation. ^j^Includes death as a cause of drug discontinuation. *HD* Haemodialysis. *PD* Peritoneal dialysis. *TNA* Treatment non-adherence

### c. Estimation of risk of bias of the included studies

**Fig. 2:**
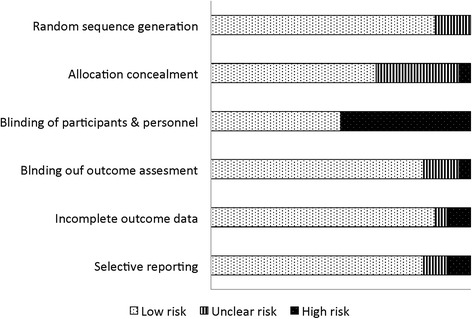
Risk of bias estimates of included trials

Was assessed using the criteria outlined in the Cochrane handbook of systematic review of interventions [33]. Random allocation was followed for each of the 22 included studies and allocation concealment was adequate in 14 of the studies (64%). Twelve studies (55%) described blinding of participants and caregivers and the blinding of outcome assessments were specified in 18 of the studies (82%). The ‘intention to treat’ principle of analysis was used in almost all of the studies (*n* = 20; 91%). Seventeen studies (77%) reported ‘loss to follow up’ data regarding outcome measurements, which ranged from 0% to 15.6% (median 0.8%). The overall risk of bias of the 22 included studies is shown in Fig. 2.

Inter-rater agreement (between authors KM and JC) was high for the study characteristics based on independently abstracted data (80.7%, *p* <0.001), which was further strengthened by consultation (95.5%, Kappa 0.88, *p* <0.001). The remaining differences were resolved by consensus among all authors.

### d. Assessment of adherence

A definition of ‘compliance’ was provided in only one of the included studies [9], which specified it as “at least 80% the scheduled intervention or placebo tablets having been taken since the previous follow-up”.

Six studies (27%) reported to have measured patient level medication adherence out of which five studies (23%) [8–10, 19, 28] provided individual adherence results. Another study [31] suggested that it measured adherence, but no results were provided. Estimates of medication possession by ‘pill count’ or verifying ‘returned drug boxes’ was used to assess adherence in all the six studies. Medication adherence was addressed to some extent in the following seven studies: four studies [11, 14, 21, 30] excluded patients who were known to be non-compliant with their medication; two studies [14, 32] reported average blood test results as an indicator of adherence rather than providing individual patient adherence; and one study [17] provided results of ‘analysis with lag censoring’ where data were censored, six months after patients discontinued the intervention (for different reasons including non-adherence).

### e. Adherence reporting and trial drug discontinuation due to potential non-adherence

Among the five studies reporting measures of individual level medication adherence [8–10, 19, 28], the reported adherence – described as the proportion of patients remaining adherent to medications - varied from between 65 to 92% in the intervention arm (mean 81%), and 82 to 87% in the control arm (mean 84.5%). In these studies, discontinuation of trial drug prior to study completion, ranged from 23 to 73% in the intervention arm (mean 37.2%) and 24 to 74% in the control arm (mean 37.2%). In one of these studies [10], that reported 92% (intervention arm) and 86% (control arm) adherence, 20% of participants in each arm discontinued medications because of “other reasons” which included “patients not willing to continue treatment” raising a suspicion of misclassification of non-adherence. Another study [19] that listed 85% adherence in the intervention and 87% in the placebo arm, reported that only 77% of the dispensed bottles were returned for pill count, raising the possibility of overestimating adherence.

Nearly all studies (21 out of 22) provided data on study drug discontinuation, with 0–73% (mean 33.2%; 95% CI, 22.0 to 44.5), in the intervention arm and 0–74% in the control arm (mean 28.8%; 95% CI, 16.8 to 40.8, in the control arm) of participants discontinuing the study medication before the completion of the trial (Refer to Table 1). The cited reasons for medication discontinuation other than adverse events, death, kidney transplantation and study drop-outs included: ‘non-adherence’ [14–17, 23]; ‘patient choice’ [8, 10, 14, 15, 17, 28]; ‘administrative reasons’ [8, 15, 17, 28]; and ‘other reasons’ [10, 14, 15] (which appear to classify elements of non-adherence in different categories).

The Consolidated Standards of Reporting of Trials (CONSORT) flow diagram depicting patient flow during the study including dropouts was provided in 91% of the studies. Out of this, three studies [9, 11, 31] did not provide data on attrition after randomization of participants in the trial flow diagram. While nine trials [8, 10, 14, 15, 17, 23, 25, 28, 30] gave different reasons for withdrawal from the study or trial drug discontinuation, only two [14, 23] studies have cited non-adherence as a reason for attrition in the CONSORT diagram.

### f. Cardiovascular/mortality outcomes and study characteristics including adherence

Thirteen studies, which included 17, 224 participants, found that the trialed medication was not significantly more effective in improving the cardiovascular or mortality outcomes when compared to the controls. On the other hand, the remaining nine studies, which included 2098 participants, found that the trialed medication did have beneficial effects when compared to the study controls (Refer to Table 1).

All four trials evaluating lipid lowering agents resulted in negative study outcomes, while studies of anti-hypertensive medications (three out of six studies positive) phosphate binding agents (two out of four trials positive) and fish oil or B-vitamins (two out of four trials positive) yielded mixed results. Two out of three trials investigating cardiac drugs including anti-platelet agents were positive, while the only large-scale trial evaluating the impact of calcimimetics on cardiovascular events was negative for the pre-specified primary outcome (Refer to Table 1).

Thirteen out of the 21 trials that provided study-drug discontinuation data, reported cessation rates of over 20%. These trials were more likely to yield a negative study outcome (11 out of 13 trials with >20% discontinuation were negative), when compared to the remaining eight trials (two out of eight trials with <20% discontinuation were negative), which had lower drug discontinuation (*p* = 0.018, Fisher’s exact test).

All the five trials that reported individual patient level adherence returned a negative result for the cardiovascular outcomes in patients with end-stage renal failure undergoing dialysis. All these studies had low risk of bias estimates for the various domains of study characteristics described in section 4 c, above. One of these trials [9] which reported advanced kidney disease and dialysis patients’ adherence data separately, showed that non-dialysis patients had an average adherence of 73%, with a higher level of adherence of 76% among a sub-set of patients with a MDRD estimated GFR over 60mls/minute. The overall study drug use were lower in the dialysis patients with an average adherence of 65%. The outcomes of this study were positive for patients who were not on dialysis and for the overall cohort, but negative for the dialysis patients.

### g. Response from authors

We contacted seventeen authors by email, when the study did not report information about measured adherence and seven authors responded. Six of the respondents had not evaluated adherence in their study, while one [11] had measured patient level adherence with an adherence of 83% for intervention and 81% for control arms. The study used ‘pill count’ as the method of measuring adherence.

## Discussion

In this systematic review, we sought to examine, how the issue of medication adherence was assessed, reported and addressed in dialysis patient trials evaluating cardiovascular or mortality outcomes. Non-adherence to therapy is important in the treatment of dialysis patients, because, at the individual patient level, it can lead to poor clinical outcomes [4] and in a clinical trial setting, a high degree of non-adherence can lead to failure to detect a true treatment effect [18]. To our knowledge this is the first systematic review, exploring the problem of non-adherence in dialysis patient trials and we have noted striking inconsistencies and inadequacies in the way in which medication adherence was reported, assessed and addressed in the eligible trials.

We noted that only 27% of the included trials have measured medication adherence to any extent and 23% reported the results of adherence based on medication possession. The low prevalence of individual patient level adherence reporting, probably reflects a failure to recognize the importance of treatment adherence as a major factor influencing clinical outcomes. It is also possible that in many of the trials cited in this review, medication adherence was actually measured, but not reported, as part of the findings. The adherence reporting, we observed in dialysis patient trials is somewhat consistent with the systematic review findings of Gossec et al. [18], evaluating the treatment adherence in six chronic diseases; namely HIV, Diabetes, Rheumatoid arthritis, Asthma, Hypertension and Osteoporosis. They found that medication adherence was assessed in 33% of the included trials, while only 25% of the trials provided results of adherence.

Consolidated Standards of Reporting Trials (CONSORT) was developed in 1996 and updated in 2001 and 2010 to improve the quality of reporting of RCTs [34]. The consensus statement has highlighted the importance of distinguishing attrition, as a result of loss to follow up, which is often unavoidable, from exclusions due to other reasons such as withdrawal from treatment and poor adherence to trial intervention [34]. The CONSORT flow diagram illustrating patient flow through the trial including components of attrition is frequently presented in publications, but the information given is often not detailed enough to ascertain the true extent and nature of non-adherence [35]. The flow diagram was provided in most of the included trials but the information was highly inconsistent making it difficult to compare between studies.

In our review, we observed that discontinuation of study medication was common but the reporting of reasons for discontinuation were not consistent between studies. The cited reasons like ‘patient choice’, ‘administrative reasons’ and ‘other reasons’ reported in the included trials appear to classify elements of non-adherence under different categories. This makes it difficult to get an accurate estimate of this problem in any given study and to compare these estimates between studies. Our review suggests that trials with a study-drug discontinuation of over 20% are more likely to yield negative study outcomes. The loss of statistical power due to high drop-out or drug discontinuation can lead to false negative outcome results [18]. In this review, since there was no consistent reporting of the causes of drug discontinuation between studies and only a small proportion of studies reported measuring adherence, we were unable to estimate the true prevalence of non-adherence and its contribution to discontinuation between studies. This made the assessment of their impact on study outcomes virtually impossible.

Some degree of non-adherence is inevitable during the conduct of any intervention trial. Addressing non-adherence can be considered in the design, conduct or analysis phase of the trial. Excluding patients who are likely to be non-adherent, is the most efficient strategy in the design phase and this was utilized by four [11, 14, 21, 30] trials included in this review. Though patients who participate in the trial may be more motivated to adhere to the prescribed treatment than those in the general population, the intensity of the trial protocol may precipitate non-adherence [35]. Liaising with the patient’s caregivers to elicit a history of poor treatment compliance has been used as a screening strategy, but the inherent difficulty in recognizing adherence in routine clinical practice may reduce its reliability. Screening during a run-in phase before randomization to unmask non-adherent behavior to exclude non-compliers, is another approach and was reported to have been used in one (8) of the studies included in this review. However, these methods are not foolproof and there is no guarantee that patients selected in this manner will remain adherent to medications throughout the RCT study period.

Efforts to increase the medication compliance during the conduct of the study in dialysis patients pose several challenges. Dialysis patients are frequently frail and chronically ill with several comorbidities and a heavy pill burden, which predispose to drop out due to trial fatigue [17]. Increasing complexity of treatment is an important factor that precipitates non-adherence [1]. These factors are highly relevant to the participants in the current systematic review.

Methods to address the effect of non-compliance in the analysis phase of the trial are prone to bias. In our review, 91% of the included trials were analyzed as “intention to treat”. When the level of non-adherence is high, the principle of assigning success or failure to an intervention, which was never received by the subject has some limitations. However, analysis by actual treatment received (TR) invalidates the assumptions underlying randomization and thereby the probabilistic meaning of reported p-values [36]. Despite this serious limitation, analysis by TR has been tried in several forms in trials where non-adherence is an issue: a) non-compliers can be counted by the treatment they actually received (‘naïve’ TR); b) non-compliers can be excluded; or c) non-compliers can be treated as censored at the time or shortly after they have stopped the treatment being tested [36]. One of the studies [17] included in our review has reported analysis with lag censoring, where data was censored six months after participants discontinued the study drug, and showed significant improvements in hazards of the primary composite outcome for the active treatment, while the ‘intention to treat’ analysis was negative. Estimators of the effect size in analysis with lag censoring, may however be biased, as analysis by TR is constrained by the same limitations as in observational epidemiology, such as confounding [36]. Nevertheless, in the setting of high trial drug discontinuation, especially for non-protocol specified reasons, such pragmatic approaches should be considered in context.

It is important to understand the difference between “efficacy”, which implies whether a specific intervention works under ideal circumstances and “effectiveness” which denotes its effect in the ‘real-world’. It could be argued that non-adherence is a "real-world" issue and in order to understand how drugs perform in the real world, it may be necessary to allow for non-adherence to occur in a clinical trial, as it occurs in usual clinical practice. However, failure to recognize and account for non-adherence in a clinical trial setting, especially when it is frequent, can mask the efficacy of the intervention being investigated. Furthermore, if the level of non-adherence is recognized to be higher than originally thought during the conduct of the clinical trial, false negative outcomes could potentially be avoided by increasing the sample size, if feasible or extending duration of study follow up.

If a specific drug is less acceptable to the patient and promotes non-adherence for this reason, its effectiveness in the ‘real-world’ is going to be lower than the “efficacy” demonstrated in a clinical trial setting.

Our study has both strengths and limitations. One of the major strengths of this review is that it is the first analysis of adherence reporting in randomized control trials evaluating cardiovascular or mortality outcomes in dialysis patients. Another strength is that our review examines the means to address the vexing problem of non-adherence in the setting of dialysis patient trials. From a limitations perspective, the number of eligible studies included in our review was small and the overall reporting of adherence was even smaller. The inconsistency in reporting of adherence and causes of trial drug discontinuation made it difficult to compare studies and combined with the varied nature of the pharmacological interventions made it meaningless to derive a pooled estimate. We would recommend the adoption of a more comprehensive and uniform approach to evaluating and reporting non-adherence in future clinical trials to assess its impact on outcomes. This should include the development of a broadly acceptable definition of non-adherence, consistent methodologies (like pill count) to measure the problem and routine reporting of measured adherence similar to other standard items reported as per CONSORT guidelines. Defining medication adherence as the intake of more than 80% of the prescribed treatment, as done by Baigent et al. [9] may be acceptable for most situations, but a blanket approach is not appropriate – for instance, drugs like anti-HIV medications and immunosuppressant drugs would warrant more stringent criteria. We also recommend a standardized approach to reporting causes of trial drug discontinuation, which will help us to compare the impact of different causes of therapy discontinuation on outcomes between different trials. Adverse events, which may or may not be related to the medication, are important causes of non-adherence and consistent reporting of the causes of non-adherence is the only way to evaluate their contribution to this problem. Considering the heterogeneous nature of the problem of adherence and treatment discontinuation, these strategies pose difficult challenges, but are nevertheless possible to achieve.

## Conclusions

In this systematic review of RCTs evaluating interventions targeted at improving clinical cardiovascular outcomes for dialysis patients, we identified inadequacies in the medication adherence reporting and inconsistencies in the reporting of causes contributing to study drug discontinuation. We also observed that the trials with high study drug discontinuation were more likely to yield negative study outcomes. We therefore recommend a more comprehensive and consistent approach to measuring and reporting adherence and the causes of study drug discontinuation in future trials, which will help to clarify the true impact of poor treatment adherence on the clinical outcomes of this vulnerable population.

## Additional file

Additional file 1: Spreadsheet detailing data extraction and synthesis. (XLSX 40 kb)

## Abbreviations

CI: Confidence interval
CKD: Chronic kidney disease
CONSORT: Consolidated standards of reporting of trials
ESKD: End stage kidney disease
GFR: Glomerular filtration rate
MDRD: Modification of diet in renal disease study
RCT: Randomized controlled trial
